# A novel antimicrobial polymer efficiently treats multidrug-resistant MRSA-induced bloodstream infection

**DOI:** 10.1042/BSR20192354

**Published:** 2019-10-15

**Authors:** Xu Chen, Weiyang Lou, Jingxing Liu, Bisha Ding, Weimin Fan, Jun Hong

**Affiliations:** 1 Department of Intensive Care Medicine, Zhejiang Provincial People’s Hospital, People’s Hospital of Hangzhou Medical College, Hangzhou, Zhejiang, China; 2 Program of Innovative Cancer Therapeutics, Division of Hepatobiliary and Pancreatic Surgery, First Affiliated Hospital, College of Medicine, Zhejiang University, Hangzhou, Zhejiang, China; 3 Department of Intensive Care Unit, Changxing People’s Hospital of Zhejiang, Huzhou, Zhejiang, China

**Keywords:** antimicrobial polymer, bloodstream infection, methicillin-resistant Staphylococcus aureus (MRSA), multidrug-resistant (MDR)

## Abstract

The present study aimed to ascertain if polymer 2a, a novel synthesized antimicrobial polyionene, could treat methicillin-resistant *Staphylococcus aureus* (MRSA)-induced bloodstream infection. The minimum inhibitory concentration (MIC) of polymer 2a against MRSA was detected. A time-kill assay was employed to determine the killing kinetic of polymer 2a. Potential antimicrobial mechanisms of polymer 2a, including membrane disruption and programmed cell death (PCD), were explored. A resistance development assay was introduced to determine the propensity of polymer 2a toward resistance against MRSA. A mouse model of MRSA bacteremia was established to assess *in vivo* efficacy of polymer 2a. Furthermore, *in vivo* toxicity of polymer 2a was also evaluated through injection by tail vein. Polymer 2a exhibited more superior antimicrobial activity and faster killing kinetic than the control antibiotics against clinically isolated MRSA strains. Polymer 2a resulted in an obvious leakage of cellular components (concentration more than 1× MIC). mRNA expression of PCD pathway-related gene (*recA*) was significantly up-regulated in the presence of polymer 2a with low concentration (concentration less than 1× MIC). Repeated use of polymer 2a did not lead to drug resistance. In a MRSA-induced bloodstream infection mouse model, polymer 2a displayed superior therapeutic efficacy with negligible systemic toxicity. Moreover, polymer 2a treatment by tail vein could evidently reduce MRSA counts in blood and major organs and markedly improve living conditions. In conclusion, all these findings presented in this work convincingly suggested that polymer 2a may be a promising therapeutic alternative for treating MRSA-induced infections, especially bloodstream infection.

## Introduction

Over the past decades, antimicrobial resistance has posed a serious threat to global health [1–3]. A group of pathogens, namely ESKAPE bacteria (*Enterococcus faecium, Staphylococcus aureus, Klebsiella pneumoniae, Acinetobacter baumannii, Pseudomonas aeruginosa, Enterobacter* species), are responsible for the majority of antibiotic resistance and hospital-acquired infections [4,5]. Among these pathogens, Gram-positive methicillin-resistant *Staphylococcus aureus* (MRSA) can cause numerous infectious diseases, such as pneumonia, pleuritis, tympanitis, purulent meningitis as well as bloodstream infection [6]. MRSA-induced bacteremia has been one of the most urgent global issues because of its high rate of treatment failure, thus leading to an overall mortality of 10–30% [7]. Such high rates of treatment failure and death may be attributed to the lack of effective therapeutic agents against MRSA-induced bloodstream infection.

It has been widely acknowledged that vancomycin is the last defense for Gram-positive pathogens, including MRSA [7]. Although patients with MRSA infections are responsive to vancomycin in general, sensitivity of MRSA toward vancomycin is inevitable [8]. Moreover, reports have demonstrated that sensitivity reduction of vancomycin is closely associated with poor outcome of patients with MRSA infection [9]. Therefore, there is an urgent need to seek and develop new effective therapeutic approaches for treating MRSA-induced infections.

To overcome this issue, scholars and researches have invested huge efforts to develop appropriate alternatives with superior antimicrobial activity and less propensity to occur resistance. Among these antimicrobial materials, antimicrobial polymers are good candidates, as they can effectively combat microbes [10]. Moreover, lots of studies have reported that antimicrobial polymers are difficult to develop resistance [11]. However, to date, high toxicity of antimicrobial peptides and polymers is still hard to address, thus impeding development from laboratory to the clinic [12]. Recently, our group have designed and synthesized a novel antimicrobial polymer, polymer 2a, which is a polyionene with rigid amide bonds along the polymer backbone [13]. Preliminary studies suggested that polymer 2a effectively and rapidly eliminated *K. pneumoniae* both *in vitro* and *in vivo*. Intriguingly, the results also demonstrated a negligible *in vivo* toxicity of polymer 2a by intraperitoneal injection.

In the present study, we intended to ascertain if polymer 2a could effectively kill MRSA and had potential in treating MRSA-induced bloodstream infection. The toxicity of polymer 2a was also determined through tail vein injection. All findings in the present study suggest that polymer 2a may be a promising therapeutic alternative for MRSA-induced infections, especially bloodstream infection.

## Materials and methods

### Bacterial strains and culture conditions

Eighteen clinically isolated multidrug-resistant (MDR) MRSA strains were extracted from blood samples of the patients which were hospitalized in the First Affiliated Hospital of Medical College, Zhejiang University (Hangzhou, China). All strains have been identified by routine laboratory methods and stored in 20% (v/v) glycerol at −80°C prior to use.

### Minimum inhibitory concentration measurements

Broth microdilution method was employed to determine the minimum inhibitory concentrations (MICs) of antimicrobial agents (including polymer 2a, ceftriaxone, levofloxacin and vancomycin) against MDR MRSA as previously described [14,15]. After grown overnight on Mueller–Hinton (MH) agar plates at 37°C, the MRSA bacteria were harvested in mid-exponential growth phase. All these antimicrobial agents were serially diluted with MHB from 1024 to 0.5 μg/ml. The bacterial suspension was diluted with phosphate-buffer saline (PBS, pH 7.4) to adjust the turbidity approximately to the standard McFarland 0.5, which nearly corresponds to the concentration of 1 × 10^8^ colony-forming unit (CFU)/ml. Then, this suspension was further diluted by 100-fold with MHB. Subsequently, 0.1 ml of the diluted bacterial suspension (1 × 10^6^ CFU/ml) was mixed with 0.1 ml of antimicrobial agent dilution, and incubated for 18 h at 37°C. Finally, the MIC was determined as the lowest concentration of these antimicrobial agents, at which no turbidity was observed with unaided eyes. Broth containing untreated MRSA bacteria was utilized as the negative control, and each experiment was performed in triplicates for three times.

### Time-kill assay

In the present study, a time-kill assay was used to evaluated the killing kinetics of polymer 2a against MRSA 25332. Preparation of bacterial suspension (1 × 10^8^ CFU/ml) was identical with MIC measurement as mentioned above. This bacterial suspension was exposed to polymer 2a, ceftriaxone, levofloxacin and vancomycin at final concentrations of 1× MIC, 2× MIC, 4× MIC and 8× MIC, and further incubated for 24 h at 37°C. A volume of 0.05 ml of samples were taken out at time points of 0, 0.5, 1.0, 1.5, 2.0, 2.5, 3.0, 3.5, 4.0, 8.0, 24.0 h, and diluted with various dilution factors with PBS. Then, each diluted bacterial suspension (0.05 ml) was plated on MH agar plates and incubated for 14 h at 37°C. Next, the number of viable colonies was counted. All experiments were performed in triplicates, and the results were presented as mean lg (CFU/ml) ± SD.

### Membrane integrity analysis

MRSA 25332 isolate was cultured overnight on MH agar plates at 37°C. Then, MRSA 25332 bacteria were suspended in PBS at the final concentration of 2 × 10^9^ CFU/ml. Polymer 2a (1/2× MIC, 1× MIC, 2× MIC, 4× MIC and 8× MIC) was added into the bacterial suspension, and incubated for 2 h at 37°C, after which the suspension was filtered with 0.22-μm filter to obtain the supernatant. The absorbance at 260 nm of the supernatant was detected by a UV spectrophotometer (Allsheng, China). Bacterial suspension without any treatment was used as the control. The experiments were performed in triplicates. The results were presented as mean ± SD.

### RNA extraction and quantitative-PCR

Total RNA was extracted from MRSA 25332 using RNAiso plus Reagent (TaKaRa, Kusatsu, Japan). Total RNA was reverse transcribed into complementary DNA (cDNA) by the PrimerScript RT Reagent Kit (TaKaRa, RR0037A). Next, SYBR Premix ExTaq (TaKaRa, RR420A) was introduced to conduct quantitative-PCR. 16S rRNA was used as the internal control. Primer sequences: *recA* forward primer, AGATCCTCTACGGCGAAGGT; *recA* reverse primer, CCTGCTTTCTCGATCAGCTT; *lexA* forward primer, GACTTGCTGGCAGTGCATAA; *lexA* reverse forward, TCAGGCGCTTAACGGTAACT; *MazEF* forward primer, CTTCGTTGCTCCTCTTGC; *MazEF* reverse primer, CGTTGGGGAAATTCACCG; *16S rRNA* forward primer, TGTAGCGGTGAAATGCGTAGA; *16S rRNA* reverse primer, CACCTGAGCGTCAGTCTTCGT. The 2^−ΔΔ*C*^_T_ method was employed to determine fold change in the RNA level. All experiments were performed in triplicates, and the results were shown as mean ± SD.

### *In vitro* drug resistant development assay

The propensity of MRSA 25332 to develop resistance against polymer 2a, ceftriaxone, levofloxacin and vancomycin was assessed using an *in vitro* drug resistant development assay [7]. MRSA 25332 isolate was successively exposed to these antimicrobial agents at sublethal doses until for 20 passages. First, MICs of these agents against MRSA 25332 were first determined using the protocols as mentioned above. This passage was defined as passage 0 (MIC_0_). Simultaneously, 20 μl of bacterial suspension from wells of 1/2× MIC were plated into MH agar plates, and cultured overnight at 37°C. MICs were also determined as previously described. The MIC values were defined as passage 1 (MIC_1_). Subsequently, using the similar approach, we further measured MICs from passages 2 to 20. The propensity of these antimicrobial agents to develop resistance was evaluated by monitoring the changes of MICs.

### Preparation of animals

ICR mice (female, 6–8 weeks, 26–30 g), kept in a specific pathogen-free environment in Laboratory Animal Center of Zhejiang University, were used for all *in vivo* experiments. Immunosuppression was induced by intraperitoneally injecting 200 mg cyclophosphamide (Hengrui Corp, Jiangsu Province, P.R. China) per kg of body weight 4 days prior to infection. All mice were anesthetized by intraperitoneal injection of 1% pentobarbital (40 mg/kg, Sigma). All animal studies were performed based on the protocols approved by the Animal Studies Committee, P.R. China.

### Mouse bloodstream infection model

A mouse bloodstream infection model caused by MRSA was used to assess the *in vivo* antimicrobial efficacy of polymer 2a. Similarly, MRSA 25332 isolates were cultured overnight at 37°C. Then, the bacteria were suspended in PBS. Each of the immunosuppressed mice was injected with 0.3 ml of the bacterial suspension at designated doses (i.e. 1 × 10^7^, 5 × 10^7^, 1 × 10^8^, 5 × 10^8^, 1 × 10^9^, 5 × 10^9^ CFU/ml, 6 mice per group) by tail vein. At 48 h post-infection, the minimal lethal dose (the lowest dose that was sufficient to cause 100% mortality) of MRSA 25332 was calculated by the BLISS method [16].

### Evaluation of 50% effective dose

The minimal lethal dose of bacterial suspension was first given to mice by tail vein. Next, various doses of polymer 2a (i.e. 0.1, 0.2, 0.4, 0.8, 1.6, 3.2, 6.4, 12.8 mg/kg, 0.2 ml per injection, 6 mice per group) and vancomycin (i.e. 0.2, 0.4, 0.8, 1.6, 3.2, 6.4, 12.8, 25.6 mg/kg, 0.2 ml per injection, 6 mice per group) were administered by tail vein at 1 and 6 h after infection. Finally, the survival rate of these treated mice was recorded over 2 days to determine 50% effective dose (ED_50_), at which 50% of infected mice are saved by treating the dose of polymer 2a/vancomycin.

### Efficacy of antimicrobial polymer in treating MRSA-induced systematic infection

Viable bacterial counts in blood and major organs (liver, spleen, kidney and lung) were also determined to further evaluate the *in vivo* efficacy of polymer 2a against MRSA. Immunosuppression and anesthesia were performed as previously mentioned. Then, each of these mice was injected with 3 × 10^7^ CFUs of MRSA 25332 (three mice per group) through tail vein. ED_50_ dose of polymer 2a or vancomycin was administered 1 and 6 h after infection by tail vein. At 48 h post-infection, three mice in each group were killed and blood and organ samples were obtained. These samples were serially diluted in PBS and plated on MH agar plates, after which these agar plates were incubated for 24 h at 37°C. At the end of incubation, the number of viable clones was counted. Infected mice without treatment of polymer 2a were used as negative control. The results were presented as mean ± SD.

To further assess the *in vivo* efficacy of polymer 2a and vancomycin against MRSA-induced bloodstream infection, survival rate of infected mice was recorded after treatment of polymer 2a and vancomycin. PBS treatment was used as the negative control. First, the immunosuppressed mice were infected with the minimal lethal dose of MRSA 25332 by tail vein. At 1 and 6 h post-infection, infected mice were administered ED_50_ dose of polymer 2a and vancomycin. All surviving mice were monitored for 2 days. Kaplan–Meier curve was analyzed using GraphPad Prism v7.

### Evaluation of 50% lethal dose

The acute hematotoxicity of polymer 2a was evaluated by measuring 50% lethal dose (LD_50_), the dose which can cause 50% mortality. The mice were first randomly assigned into eight groups (six mice per group). Then, polymer 2a was dissolved in PBS and administered to the mice at designated doses for each group (i.e. 10, 20, 30, 40, 50, 60, 70 and 80 mg/kg) by tail vein. The surviving mice were monitored for 2 days post-treatment. The value of LD_50_ was calculated by the method of BLISS [16].

### Determination of toxicity of polymer 2a toward major organs

In addition to acute toxicity, we also determined the toxicity of polymer 2a toward major organs, including liver and kidney. Simply, the mice were also randomly assigned into two groups (PBS control group and polymer 2a-treated group). Each of mice in polymer 2a-treated group received an injection of polymer 2a at its ED_95_ dose twice. At 7 days post-treatment, each of the mice was killed and blood samples were acquired from periorbital plexus. Then, these blood samples were sent to the First Affiliated Hospital, Zhejiang University (Hangzhou, China) for analysis of alanine aminotransferase (ALT), aspartate aminotransferase (AST), creatinine, urea nitrogen, sodium and potassium ion concentrations. The results were shown as mean ± SD.

### Statistical analysis

Minimum lethal dose of MRSA 25332, ED_50_ and LD_50_ of polymer 2a were calculated using the BLISS method. Differences between two groups were estimated by Student’s *t* test using GraphPad Prism v7. GraphPad Prism v7 was also employed to conduct survival analysis based on a log-rank test. *P*-value <0.05 was considered as statistically significant.

## Results

### *In vitro* antimicrobial activities of polymer 2a against MRSA

We previously reported several antimicrobial polymers, among which polymer 2a possessed potent *in vitro* and *in vivo* antimicrobial activity in treating MDR *K. pneumoniae* lung infection with negligible systemic toxicity [13]. Previous results also demonstrated that the MIC value of polymer 2a against MRSA 25312 was only 8 μg/ml [13]. In the present study, we aimed to investigate if polymer 2a could effectively treat bacteremia or systemic infection caused by MRSA.

We first determined MICs to ascertain the *in vitro* antimicrobial activity of polymer 2a against 18 clinically isolated MRSA strains. Two conventional first-line antibiotics (ceftriaxone and levofloxacin) and one last-line antibiotic (vancomycin) was employed as three control antibiotics. The MIC values of polymer 2a and control antibiotics against these MRSA strains were listed in Table 1. Table 1 revealed that polymer 2a was more potent than both ceftriaxone and levofloxacin in inhibiting growth of clinically isolated MDR MRSA isolates. At the MIC value of 16 μg/ml, polymer 2a could inhibit growth 100% isolates whereas none of isolates was suppressed by ceftriaxone and levofloxacin. In view of MIC_50_ (i.e. MIC at which 50% of isolates tested are inhibited), polymer 2a was also more effective than both ceftriaxone and levofloxacin, with MIC_50_ of 8 versus 256 and 64 μg/ml, respectively. For vancomycin, relative low MIC values were observed, with MICs ranging from 0.5 to 4 μg/ml. However, according to Clinical and Laboratory Standards Institute, MIC value of 2 μg/ml is the break point between the sensitive and intermediate states [17,18]. Therefore, 12 out of 18 MRSA isolates were not sensitive to vancomycin.

**Table 1:** Cumulative distribution of MIC value (μg/ml) against clinically isolated MRSA strains (*n*=18)

Agents	Cumulative % of 20 MRSA strains at indicated MICs										
	0.5	1	2	4	8	16	32	64	128	256	≥512
Polymer 2a			5.6	27.8	94.4	100					
Ceftriaxone								11.1	27.8	83.3	100
Levofloxacin							22.2	61.1	83.3	94.4	100
Vancomycin	11.1	33.3	77.8	100							

**Figure 1:**
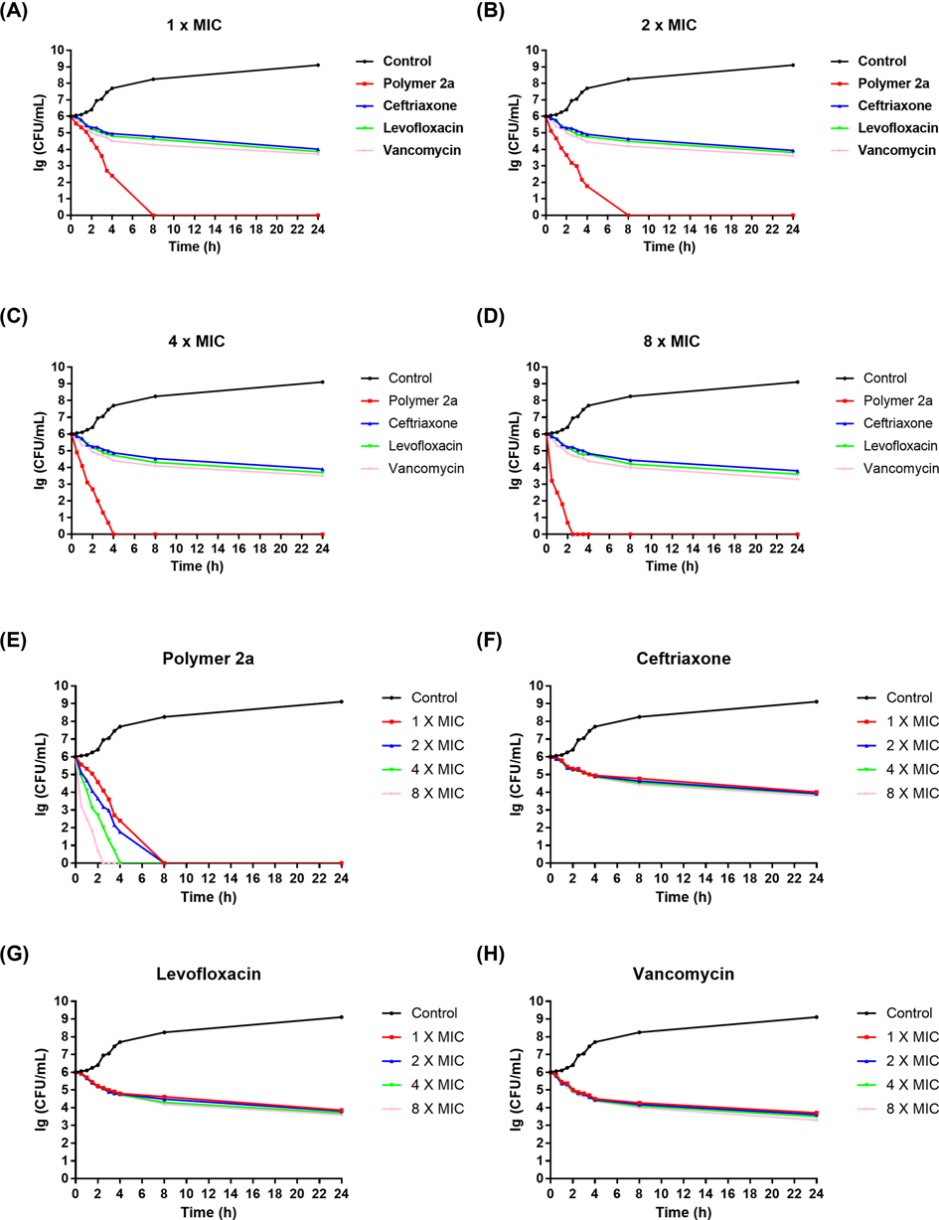
*In vitro* killing kinetics of polymer 2a, ceftriaxone, levofloxacin and vancomycin against MRSA 25332 CFUs of MRSA 25332 after treatment at different time points and various concentrations: (**A**) 1× MIC, (**B**) 2× MIC, (**C**) 4× MIC and (**D**) 8× MIC. Effect of different concentrations of polymer 2a (**E**), ceftriaxone (**F**), levofloxacin (**G**) and vancomycin (**H**) on killing kinetics. Error bars indicate respective standard deviation (s.d.) for *n*=3.

To further determine the bactericidal effect of polymer 2a against MRSA, a time-kill assay was conducted. MRSA 25332 isolate was employed as the representative strain. Ceftriaxone, levofloxacin and vancomycin were also used as the control antibiotics. MICs of polymer 2a, ceftriaxone, levofloxacin and vancomycin were measured and displayed in Table 2. Against this particular MRSA isolate, polymer 2a also showed superior efficacy than ceftriaxone and levofloxacin, with MIC value of 4 versus 256 and 64 μg/ml, respectively. Killing kinetics of polymer 2a, ceftriaxone, levofloxacin and vancomycin are presented in Figure 1. Figure 1A–D revealed that polymer 2a killed the bacteria faster than the other control antibiotics at concentrations of 1× MIC, 2× MIC, 4× MIC and 8× MIC. Figure 1E revealed that polymer 2a displayed a dose-dependent bactericidal effect. For example, 4 h were required for polymer 2a to completely eliminate all MRSA bacteria at 4× MIC while only 2.5 h were needed to do so at 8× MIC. No dose-dependent effect was observed for ceftriaxone (Figure 1F), levofloxacin (Figure 1G) and vancomycin (Figure 1H).

**Table 2:** MIC values (μg/ml) of antimicrobial agents against MRSA25332

Strain	Antimicrobial agents	MIC
MRSA 25332	Polymer 2a	4
	Ceftriaxone	256
	Levofloxacin	64
	Vancomycin	2

### Antimicrobial mechanism study

**Figure 2:**
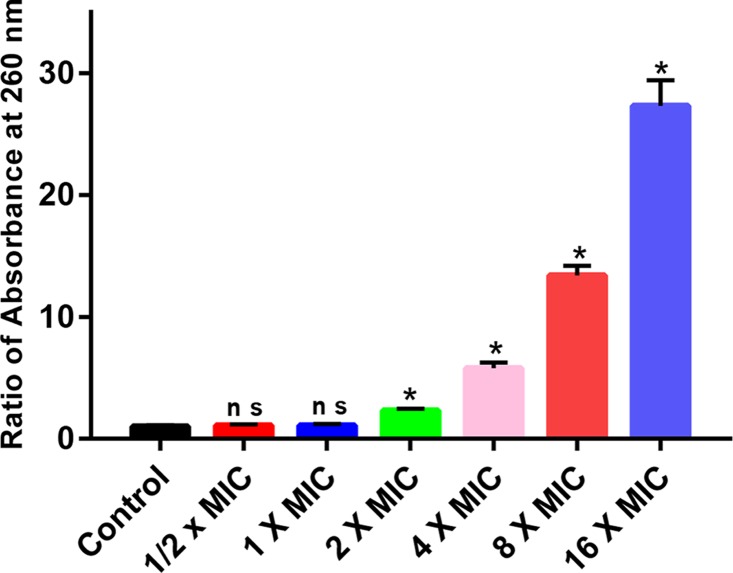
Membrane-disruption antimicrobial mechanism of polymer 2a Absorbance at 260 nm of the supernatants of MRSA 25332 treated with PBS or polymer 2a at concentrations of 1/2× MIC, 1× MIC, 2× MIC, 4× MIC, 8× MIC and 16× MIC. ns, not significant. *P<0.05. Error bars represent standard deviation (s.d.) for *n*=3.

Next, we intended to explore the antimicrobial mechanism of polymer 2a in overcoming MRSA. As nucleic acids have the highest absorption at 260 nm, we performed a membrane integrity analytic assay by recording the absorbance at 260 nm of bacterial supernatants (Figure 2). When compared with negative control, no significant differences were found in the supernatants of bacteria treated by polymer 2a at concentrations of 1/2× MIC or 1× MIC. However, leakage of the cytoplasmic materials was obviously increased when concentrations of polymer 2a were at 2× MIC, 4× MIC, 8× MIC and 16× MIC. We also found that the leakage of cytoplasmic contents by polymer 2a was concentration-dependent, which was identical with its killing kinetic we observed in the time-kill assay.

**Figure 3:**
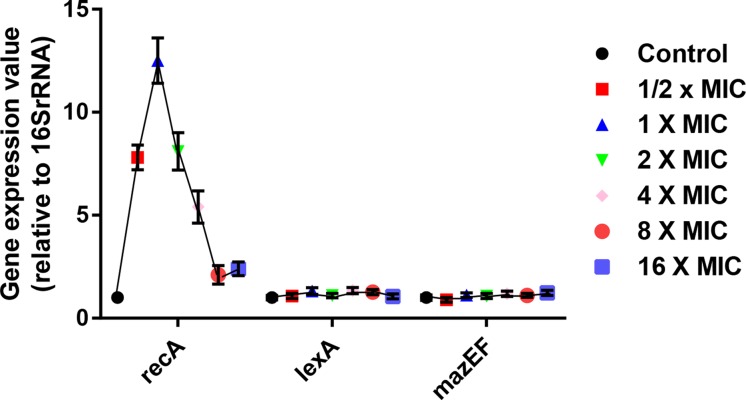
Gene expression levels of PCD-associated genes after treatment of various concentrations of polymer 2a at 1/2× MIC, 1× MIC, 2× MIC, 4× MIC, 8× MIC and 16× MIC Error bars represent s.d. for *n*=3.

Certain individual cells of multicellular organisms die by inherent mechanisms called as programmed cell death (PCD), which was also reported to kill bacteria [19]. To date, two major PCD pathways, including the ALD pathway mediated by *recA* and *lexA* genes and the mazEF pathway mediated by *mazEF* gene, have been described in bacteria [20]. Then, we detected expression changes of PCD-associated genes (*recA, lexA* and *mazEF*) by qRT-PCR (Figure 3). Figure 3 demonstrated that *recA* expression was gradually increased at polymer 2a concentrations from 1/2× MIC to 1× MIC but was rapidly decreased at polymer 2a concentrations from 1× MIC to 16× MIC. At 1× MIC, *recA* expression level reached the highest value. For *lexA* and *mazEF*, gene expression levels were not changed with the concentration changes of polymer 2a. These findings suggested that polymer 2a may cause PCD of MRSA via regulation of ALD pathway by increasing *recA* expression, especially at low dosage of polymer 2a (such as 1× MIC).

### Polymer 2a resistance development study

**Figure 4:**
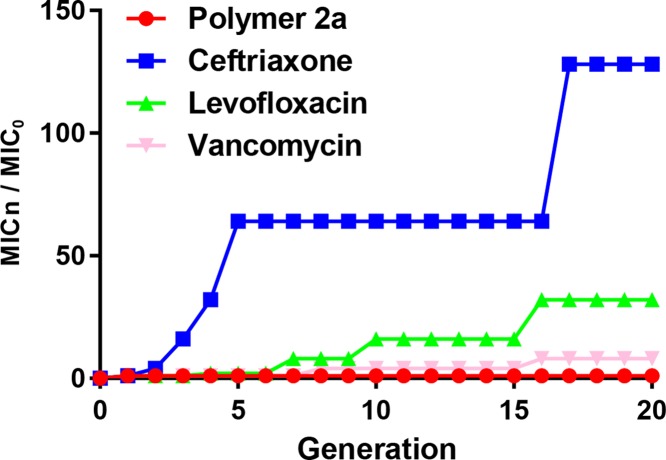
Assessment of the bacterial resistance development in MRSA 25332 by repeated exposure toward polymer 2a, ceftriaxone, levofloxacin and vancomycin at sublethal concentrations for 20 passages

Repeated exposure of bacteria to antibiotics often results in the development of drug resistance [21]. To evaluate the propensity of MRSA to develop resistance toward polymer 2a, we conducted an *in vitro* resistance development study. MRSA 25332 was used as the representative strain. Similarly, ceftriaxone, levofloxacin and vancomycin were employed as the control antibiotics. As presented in Figure 4, the MICs of polymer 2a against MRSA 25332 were not changed over the entire 20 passages. However, the control antibiotics displayed drug resistance at varying degrees. For example, MIC values of ceftriaxone, levofloxacin and vancomycin started to increase by the 3rd, 7th and 8th passages, respectively. By the 20th passage, the MIC values of ceftriaxone, levofloxacin and vancomycin increased by 128, 32 and 8 times, respectively. All these findings demonstrate that there is a lower propensity of MRSA to develop resistance toward polymer 2a than ceftriaxone, levofloxacin and vancomycin.

### *In vivo* efficacy of polymer 2a against bloodstream infection induced by MRSA

**Figure 5:**
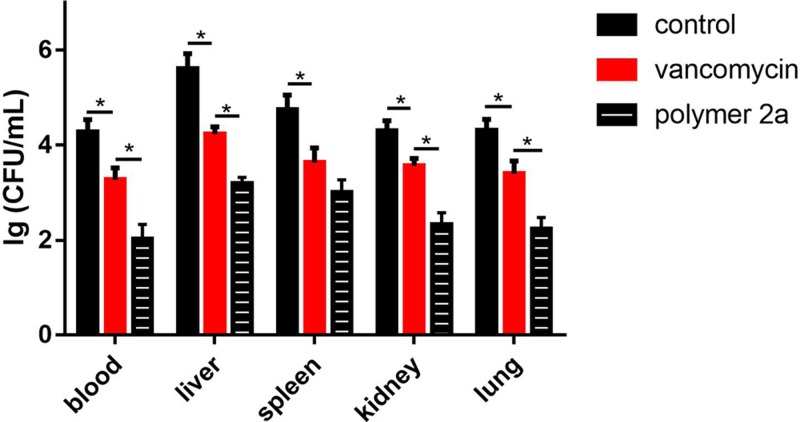
The number of viable bacterial colonies in the blood, liver, spleen, kidney and lung specimens taken from polymer 2a-treated, vancomycin-treated or PBS-treated mice at 48 h post-infection *P<0.05. Error bars represent s.d. for *n*=3.

**Figure 6:**
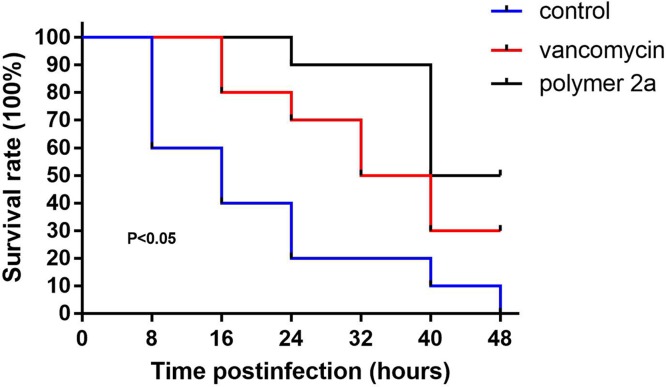
Kaplan–Meier survival curves of the infected mice (ten mice per group) Each of immunosuppressed mice were injected with 3 × 10^7^ CFUs by tail vein. Then, polymer 2a/vancomycin at concentration of ED_50_ and PBS were administered 1 and 6 h post-infection by tail vein. The surviving mice were monitored for 48 h.

In the present study, we assessed the *in vivo* efficacy of polymer 2a and vancomycin by measuring ED_50_ and ED_95_ (effective doses that lead to survival of 50% and 95% infected mice, respectively) in a mouse bloodstream infection model induced by MRSA 25332. As listed in Table 3, polymer 2a (ED_50_ of 1.13 mg/kg and ED_95_ of 5.17 mg/kg) possessed superior therapeutic effect than vancomycin (ED_50_ of 5.74 mg/kg and ED_95_ of 30.41 mg/kg). Furthermore, after treatment of polymer 2a, the bacterial burden in blood and organs from infected mice was significantly reduced when compared with vancomycin-treated mice. The results are presented in Figure 5. In the blood, mice with polymer 2a treatment had a 2.25 log_10_ reduction in bacterial load compared with PBS-treated group. However, mice with vancomycin treatment only had 0.82 log_10_ reduction in bacterial load compared with PBS-treated group. Bacterial burden of other organs in polymer 2a-treated group was also lower than that in vancomycin-treated group. Besides, survival rate of infected mice after treatment of polymer 2a or vancomycin was determined as presented in Figure 6. Figure 6 demonstrated that polymer 2a treatment could significantly prolong survival time and improve outcome of infected mice when compared with PBS-treated mice or vancomycin-treated mice. For example, at time point of 24 h post-infection, eight out of ten mice were dead in the control group, but seven out of ten and nine out of ten mice were still alive in vancomycin-treated group and polymer 2a-treated group, respectively. Taken together, polymer 2a has a potential in treating MRSA-induced bloodstream infection.

**Table 3:** *In vivo* efficacy of polymer 2a against a MRSA-induced systemic infection mouse model (*n*=6 in each group)

Antimicrobial agent	Polymer 2a	Vancomycin
ED_50_ (mg/kg)	1.13	5.74
ED_5_ (mg/kg)	0.25	1.08
95% confidence interval (mg/kg)	2.49–5.13	3.28–10.85
ED_95_ (mg/kg)	5.17	30.41

### *In vivo* toxicity of polymer 2a

First, we determined the acute toxicity of polymer 2a (injection by tail vein) by detecting 5% lethal dose (LD_5_), median lethal dose (LD_50_) and 95% lethal dose (LD_95_). As listed in Table 4, polymer 2a displayed a low acute toxicity, with LD_50_ of 31.38 mg/kg. The *in vivo* therapeutic index (LD_50_/ED_50_) of polymer 2a was 27.8 (31.38/1.13), which is much higher than other antimicrobial polymers developed by our team and other labs, implying a relatively satisfactory safety of polymer 2a [7].

**Table 4:** *In vivo* toxicity of polymer 2a treatment via tail vein (*n*=6 in each group)

Antimicrobial agent	Polymer 2a
LD_50_ (mg/kg)	31.38
LD_5_ (mg/kg)	10.82
95% confidence interval (mg/kg)	20.49–41.37
LD_95_ (mg/kg)	91.02

Next, we further evaluated the potential toxicity of polymer 2a toward major organs and blood sodium/potassium ion concentrations. Polymer 2a-treated mice received an injection of polymer 2a at dosage of ED_95_ twice by tail vein. Intriguingly, no significant changes were observed between polymer 2a-treated group and control group in the serum levels of ALT, AST, creatinine, urea, sodium ion and potassium ion (Table 5). The findings reveal that polymer 2a possesses negligible *in vivo* toxicity.

**Table 5:** Liver function, kidney function and blood sodium/potassium ion concentration of mice after polymer 2a (ED_50_ dosage for twice) and PBS treatment via tail vein (*n*=6 in each group)

	ALT^1^ (U/l)^2^	AST^3^ (U/l)	Creatinine (mmol/l)	Urea nitrogen (mmol/l)	Sodium ion (mmol/l)	Potassium ion (mmol/l)
PBS	28.0 (3.8)	88.1 (5.2)	17.8 (3.3)	8.5 (1.9)	141.9 (5.0)	4.2 (0.7)
Polymer 2a	31.0 (4.1)	91.6 (7.9)	16.9 (4.0)	9.4 (2.0)	144.5 (7.2)	4.6 (0.5)
Polymer 2a vs. PBS	*P*>0.05	*P*>0.05	*P*>0.05	*P*>0.05	*P*>0.05	*P*>0.05

1 ALT, alanine transaminase.

2 U/l, international units per liter.

3 AST, aspartate transaminase.

## Discussion

ESKAPE bacteria are the leading cause of severe infections with limited or no effective antimicrobial choices because of antimicrobial resistance [22]. Among these infections, MRSA-induced bloodstream infection is an extremely lethal health threat. Due to the response decrease toward vancomycin and other antimicrobial options, MRSA-induced bloodstream infection and subsequent systemic infection is one of the most important public health issues globally and is a difficult problem for clinicians. Therefore, it is urgent and meaningful to rapidly seek and develop effective therapeutic approaches against MRSA.

Over the past decades, a variety of antimicrobial agents have been described. Antimicrobial polymers are one of the most promising materials for treating infectious diseases. Though the potent therapeutic effect, most of antimicrobial polymers present high toxicity, which is the main reason that impedes development from laboratory to the clinic. By optimizing structure and size, our team designed and synthesized a novel antimicrobial agent, polymer 2a. In previous study, we reported that polymer 2a was a therapeutic choice for treatment of MDR *K. pneumoniae*-caused lung infection [13]. In this work, we aimed to evaluated the *in vitro* and *in vivo* antimicrobial efficacy of polymer 2a in treating MRSA. Moreover, the action mechanism and toxicity research of polymer 2a was also explored.

Initially, we determined the potent *in vitro* antimicrobial effect of polymer 2a by measuring MIC value, comparing with three clinically used antibiotics, namely ceftriaxone, levofloxacin and vancomycin. Then, *in vitro* time-killing assay revealed that polymer 2a could rapidly eliminate MRSA, with dose-dependent performance. This potent and rapid dose-dependent antimicrobial character is important for its development of clinically used antimicrobial agent.

Next, we preliminarily explored the antimicrobial mechanisms of polymer 2a in treating MRSA. Previous studies from our team and other labs have well documented that antimicrobial polymers kill pathogens by rapidly forming pores on the plasma, thus leading to the leakage of cytoplasmic contents (including nucleic acids and proteins) and finally cell death [13,15,17]. To ascertain if polymer 2a function by a similar antimicrobial approach, leakage of intracellular content was first analyzed. The leakage was dramatically up-regulated when concentration of polymer 2a was more than 1× MIC. However, polymer 2a with low concentration, such as 1/2× MIC and 1× MIC, show no significant increase in content leakage. Several studies have also reported that two major PCD pathways, including the ALD pathway mediated by *recA* and *lexA* genes and the *mazEF* pathway, are involved in killing bacteria [20,23,24]. By determining expression changes of *recA, lexA* and *mazEF*, we discovered that *recA* expression was markedly increased at polymer 2a concentrations from 1/2× MIC to 1× MIC but was rapidly decreased at polymer 2a concentrations from 1× MIC to 16× MIC. Taken together, we speculate that there are two potential killing mechanisms of polymer 2a against MRSA. PCD may dominate at low dosage of polymer 2a but membrane disruption may be the more important action mechanism at high dosage of polymer 2a.

Extensive reasons account for antimicrobial resistance, among which repeated exposure of bacteria to antibiotics at sublethal concentrations, including Gram-positive MRSA [13,15]. Therefore, in the present study, we evaluated the propensity of polymer 2a to develop resistance against MRSA. The result demonstrated that no resistant development was observed, whereas the control antibiotics presented various degrees of drug resistance. These findings together show that polymer 2a is a promising alternative for overcoming antimicrobial resistance, especially MRSA.

The superior *in vitro* antimicrobial activity of polymer 2a against MRSA pushed us to further study the *in vivo* antimicrobial efficacy of polymer 2a. MRSA can cause multiple infectious diseases, among which bloodstream infection is one of the most lethal and intractable in the clinical settings. Therefore, a MRSA-induced bloodstream infection model was employed to assess the *in vivo* antimicrobial effect of polymer 2a. Vancomycin was used as the control antibiotic. First, by determining ED_50_ and ED_95_, polymer 2a present a superior therapeutic effect than vancomycin in this model. Moreover, administration of polymer 2a significantly reduced the bacterial burden of blood and organs and prolong survival time and improve outcome of infected mice when compared with vancomycin-treated group and PBS-treated group.

*In vivo* toxicity of antimicrobial agents, including antimicrobial polymers and peptides, greatly limit their clinical usage [25]. Thus, assessment of *in vivo* toxicity of polymer 2a is critical for its application in the future. Our current study suggested that polymer 2a cause negligible acute and chronic toxicity by determining therapeutic index, ALT, AST, creatinine, urea, sodium ion and potassium ion. Taken together, polymer 2a may be a promising candidate for treatment of MRSA-caused infection, especially bloodstream infection.

## Conclusions

This research demonstrates that polymer 2a possesses more potent *in vitro* antimicrobial activity against MRSA with lower MIC values than clinically used antibiotics. It also kills bacteria much faster than the control antibiotics with lower propensity for resistance development. Furthermore, polymer 2a displays a superior *in vivo* efficacy than vancomycin in treating bloodstream infection induced by MRSA. In particular, the therapeutic dosage of polymer 2a does not cause significant toxicity. Combining potential therapeutic effect and negligible toxicity, polymer 2a may be utilized to treat MRSA-caused infection, especially bloodstream infection.

## Abbreviations

ALT: alanine aminotransferase
AST: aspartate aminotransferase
CFU: colony-forming unit
ED_50_: 50% effective dose
ESKAPE: *Enterococcus faecium, Staphylococcus aureus, Klebsiella pneumoniae, Acinetobacter baumannii, Pseudomonas aeruginosa, Enterobacter* species
ICR: institute of cancer research
LD_50_: 50% lethal dose
MDR: multidrug-resistant
MH: Mueller–Hinton
MIC: minimum inhibitory concentration
MIC_50_: MIC at which 50% of isolates tested are inhibited
MRSA: methicillin-resistant *Staphylococcus aureus*
PBS: phosphate-buffer saline
PCD: programmed cell death
SD: standard deviation
